# The nature of ancient Egyptian copper-containing carbon inks is revealed by synchrotron radiation based X-ray microscopy

**DOI:** 10.1038/s41598-017-15652-7

**Published:** 2017-11-10

**Authors:** Thomas Christiansen, Marine Cotte, René Loredo-Portales, Poul Erik Lindelof, Kell Mortensen, Kim Ryholt, Sine Larsen

**Affiliations:** 1 Department of Cross-Cultural and Regional Studies (ToRS), Section of Egyptology, University of Copenhagen, Karen Blixens Plads 8, 2300 Copenhagen S, Denmark; 2 European Synchrotron Radiation Facility (ESRF) 71 avenue des Martyrs CS 40220, 38043 Grenoble Cedex 9, France; 3 Sorbonne Universités, UPMC Univ Paris 06, CNRS, UMR 8220, Laboratoire d’archéologie moléculaire et structurale (LAMS), 4 Place Jussieu, 75005 Paris, France; 4 National Council on Science and Technology (CONACyT), National Autonomous University of Mexico, Geology Institute, Regional Northwest Station, Av. Luis Donaldo Colosio, 83000 Hermosillo, Sonora Mexico; 5 Niels Bohr Institute (NBI), University of Copenhagen, Universitetsparken 5, 2100 Copenhagen Ø, Denmark; 6 Department of Chemistry, University of Copenhagen, Universitetsparken 5, 2100 Copenhagen Ø, Denmark; 7 Present Address: Fondazione Museo delle Antichità Egizie di Torino, Via Accademia delle Scienze 6, 10123 Turin, Italy

## Abstract

For the first time it is shown that carbon black inks on ancient Egyptian papyri from different time periods and geographical regions contain copper. The inks have been investigated using synchrotron-based micro X-ray fluorescence (XRF) and micro X-ray absorption near-edge structure spectroscopy (XANES) at the European Synchrotron Radiation Facility (ESRF). The composition of the copper-containing carbon inks showed no significant differences that could be related to time periods or the geographical locations. This renders it probable that the same technology for ink production was used throughout Egypt for a period spanning at least 300 years. It is argued that the black pigment material (soot) for these inks was obtained as by-products of technical metallurgy. The copper (Cu) can be correlated with the following three main components: cuprite (Cu_2_O), azurite (Cu_3_[CO_3_]_2_[OH]_2_) and malachite (Cu_2_CO_3_[OH]_2_).

## Introduction

Two of the most profound technological advances in human intellectual history were the twin inventions of ink and papyrus, the ancient precursor of modern paper, by the Egyptians about 5.000 years ago. The advent of writing allowed information to be expanded beyond the mental capacity of any single individual and to be shared across time and space. The two inventions spread throughout the ancient Mediterranean to Greece, Rome and beyond. The chemistry of the black inks used in the ancient world has been only scantily studied so far, leaving gaps in our knowledge of one of the fundamental inventions in the history of civilization^1^. Thus, until recently, it was assumed that the ink used for writing was primarily carbon-based at least until the 4^th^ to the 5^th^ century CE. However, micro XRF analyses of two papyrus fragments from Herculaneum have shown that lead compounds were added to black ink already in 1^st^ century CE, thereby modifying our knowledge of ink manufacture in Antiquity^2,3^.

Here, we report on the chemical composition of black ink inscribed on papyrus fragments from ancient Egypt using micro XRF and XANES. The fragments form parts of larger manuscripts belonging to the Papyrus Carlsberg Collection, University of Copenhagen, and can be divided into two groups:

The first group comes from southern Egypt and consists of the private papers of an Egyptian soldier, Horus, who was stationed at the military camp of Pathyris, located at modern Gebelein some 30 km south of Luxor. Pathyris was destroyed in 88 BCE during a civil war and thousands of papyri have been preserved in the ruins until modern times and are now conserved in papyrus collections around the world, including Berlin, Cairo, Heidelberg and Turin, as well as Copenhagen. Our archive consists of 50 Greek and Egyptian papyri that date to the late 2^nd^ and early 1^st^ century BCE. They were bought on the antiquities market in 1924 by the manuscript collector Elkan Nathan Adler (1861–1941) according to whom they had been found inside a sealed jar at the ancient settlement^4^. This is the only archive from Pathyris that have come down to posterity substantially intact^5^.

The second group derives from the only large scale institutional library to survive from ancient Egypt, the Tebtunis temple library. The assemblage includes an estimated 400–500 papyrus manuscripts which span the 1^st^ through the early 3^rd^ century CE, with the bulk dating to the late 1^st^ and 2^nd^ centuries. It was discovered within two small cellars inside the main temple precinct at Tebtunis, modern Umm el-Breigât, which is located in the south of the Fayum depression, some 100 km south-west of Cairo. The dry and brittle manuscripts are all poorly preserved and the material as a whole now consists of many thousands of smaller fragments, which are preserved in papyrus collections around the world, including Copenhagen, Florence, Berlin, Berkeley, Oxford and Yale. Whole columns or pages are only rarely preserved, and the difficult and time consuming process of sorting and identifying fragments of specific manuscripts is still ongoing. Published texts indicate that on average less than 10% of a manuscript is likely to have been preserved. The papyri selected for analysis were acquired for the Papyrus Carlsberg Collection between 1931 and 1938 on the antiquities market in Cairo^6^.

Recently, the chemical composition of papyri and ink from the two localities was studied using a combination of laboratory XRF point analysis, Raman spectroscopy and scanning electron microscopy-energy dispersive x-ray spectroscopy (SEM-EDXS). Despite their distance in time, space, and social context, the study concluded that the black inks of Pathyris and Tebtunis revealed similar traits and that – besides carbon ink – two other distinct types of black ink were used for at least a period of 300 years: lead-containing carbon ink and copper-containing carbon ink. However, this preliminary characterization was limited to conventional XRF (few points), Raman and SEM-EDXS (small area maps) techniques and the chemical nature of the lead (Pb) and copper (Cu) compounds detected in the black inks could not be ascertained through the experimental setup^7^.

## Experimental

### Samples

In total, the research was conducted on a corpus of 12 fragments. The papyri are of a light brown color and the inks range from deep black to light grey or brown (cf. the visible light pictures shown in the figures). The papyrus medium itself is approximately 0.3 mm thick and made of two layers of papyrus strips – in one instance, where two sheets overlap, of four layers (sample 1).

**Figure 1:**
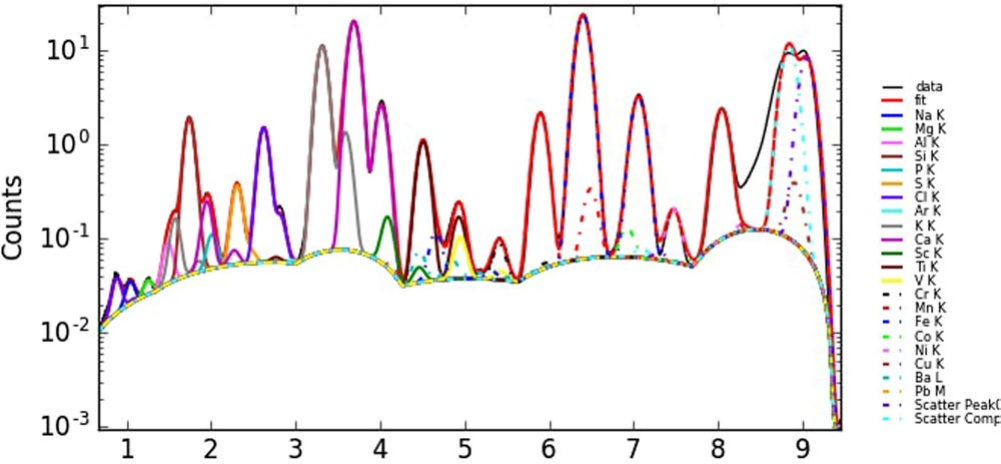
Example of a XRF fit (sample 1).

The macro XRF elemental maps, discussed below, showed either no contrast between the inked areas and the papyrus, indicating soot or finely powdered charcoal as the origin of the black color, or the presence of Cu or Pb compounds in the pigments. In Fig. 1, an example of a XRF fit is shown, which demonstrates that the main elements can be identified with certainty.

Out of the 12 samples, five showed no contrast, six contained Cu and a single fragment Pb. Here, we report results obtained from a study of four samples with Cu-containing black inks, two from Pathyris and two from Tebtunis respectively. The four samples were chosen, because they showed an intense Cu signal in the inked areas. Further, as an example (sample 5), a carbon based ink from Pathyris is included in the supporting information (Fig. S1).

**Figure 2:**
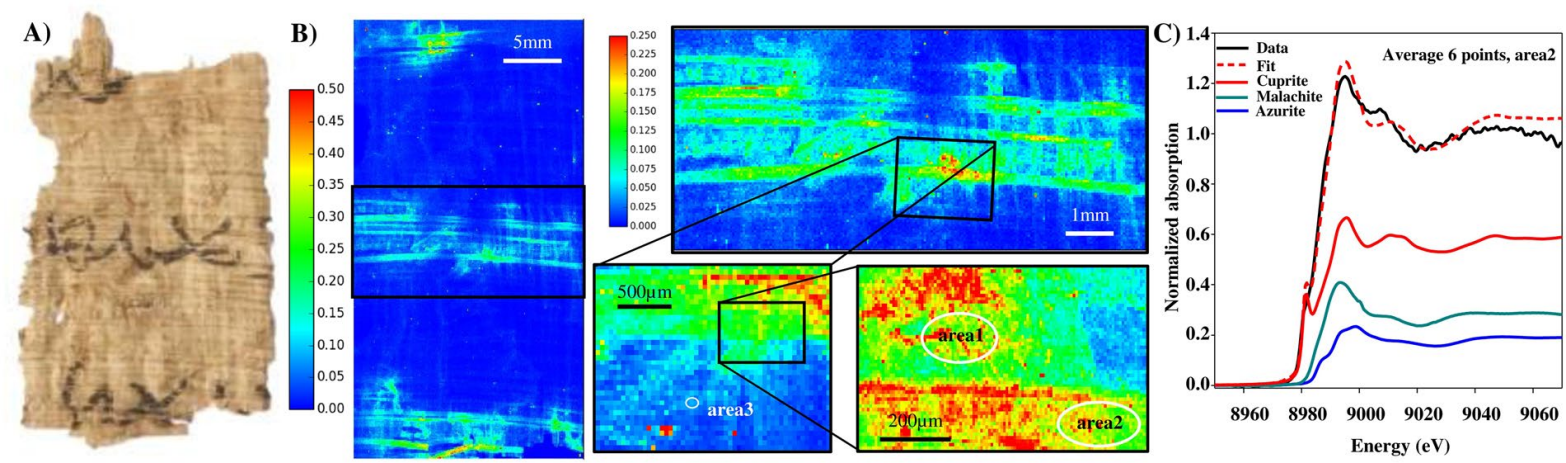
(**A**) Visible light picture of sample 1 (P. Carlsberg 828) (**B**) macro and micro XRF maps of Cu (fitted and normalized by the intensity of incident beam). The areas were XANES spectra were collected are highlighted (**C**) Average XANES spectra from area 2, and its decomposition by LCF.

**Figure 3:**
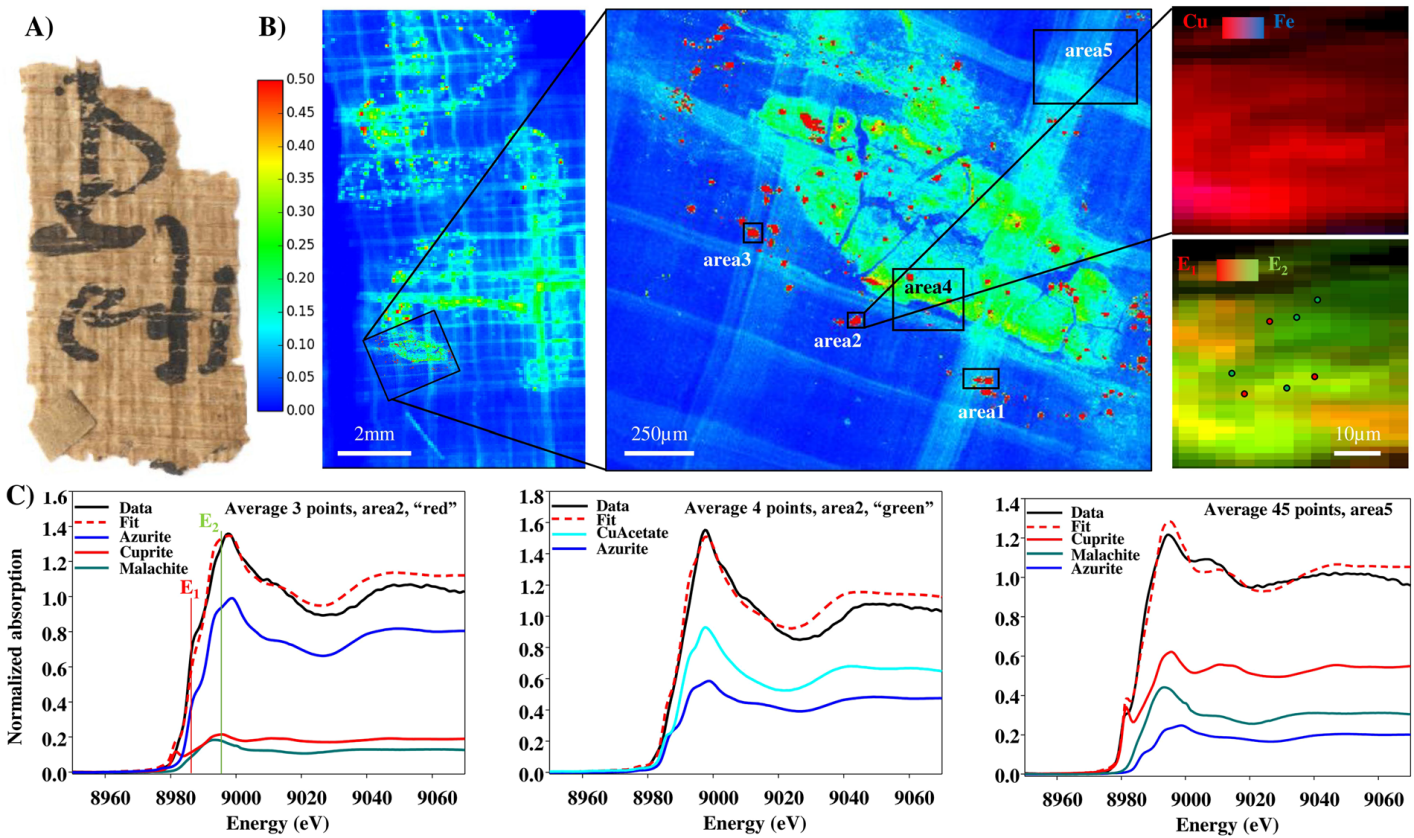
(**A**) Visible light picture of sample 2 (P. Carlsberg 839) (**B**) macro and micro XRF maps of Cu (fitted and normalized by the intensity of incident beam). The areas were XANES spectra were collected are highlighted. The red-blue maps are the superimposition of Cu and Fe maps, from area 2. The red-green maps are the superimposition of Cu micro XRF maps excited at two specific energies shown in (**C**) (after realignment) (**C**) Average XANES spectra from area 2, “red region” and “green region” in red-green dual-energy map, and from area 5 and their decomposition by LCF.

**Figure 4:**
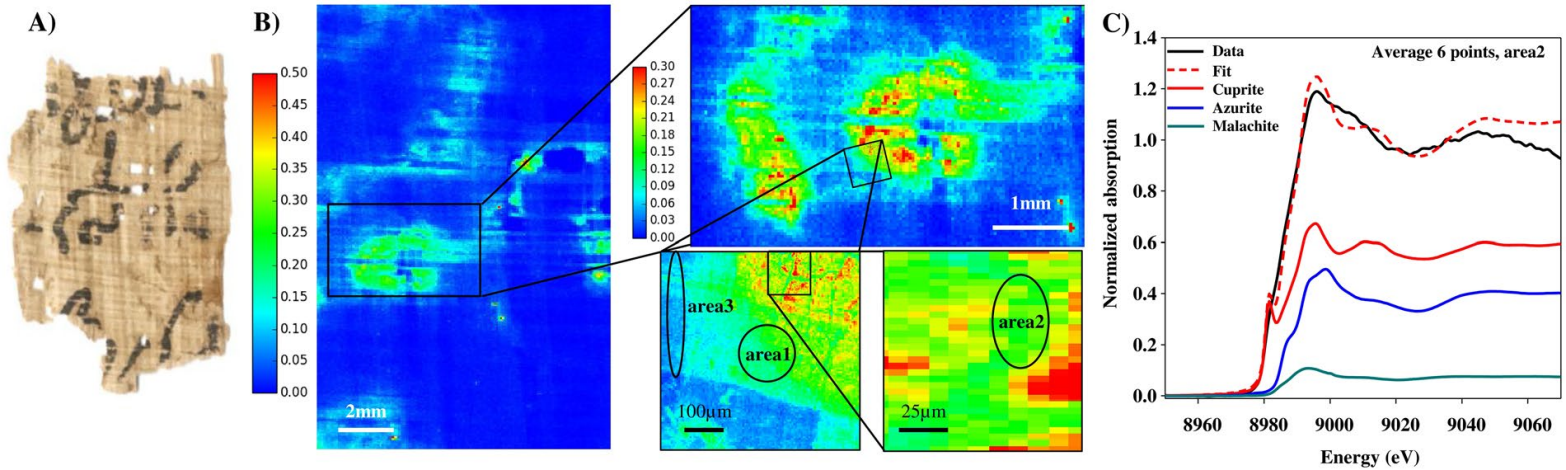
(**A**) Visible light picture of sample 3 (P. Carlsberg 79) (**B**) macro and micro XRF maps of Cu (fitted and normalized by the intensity of incident beam). The areas, where XANES spectra were collected, are highlighted (**C**) Average XANES spectra from area 2 and its decomposition by LCF.

**Figure 5:**
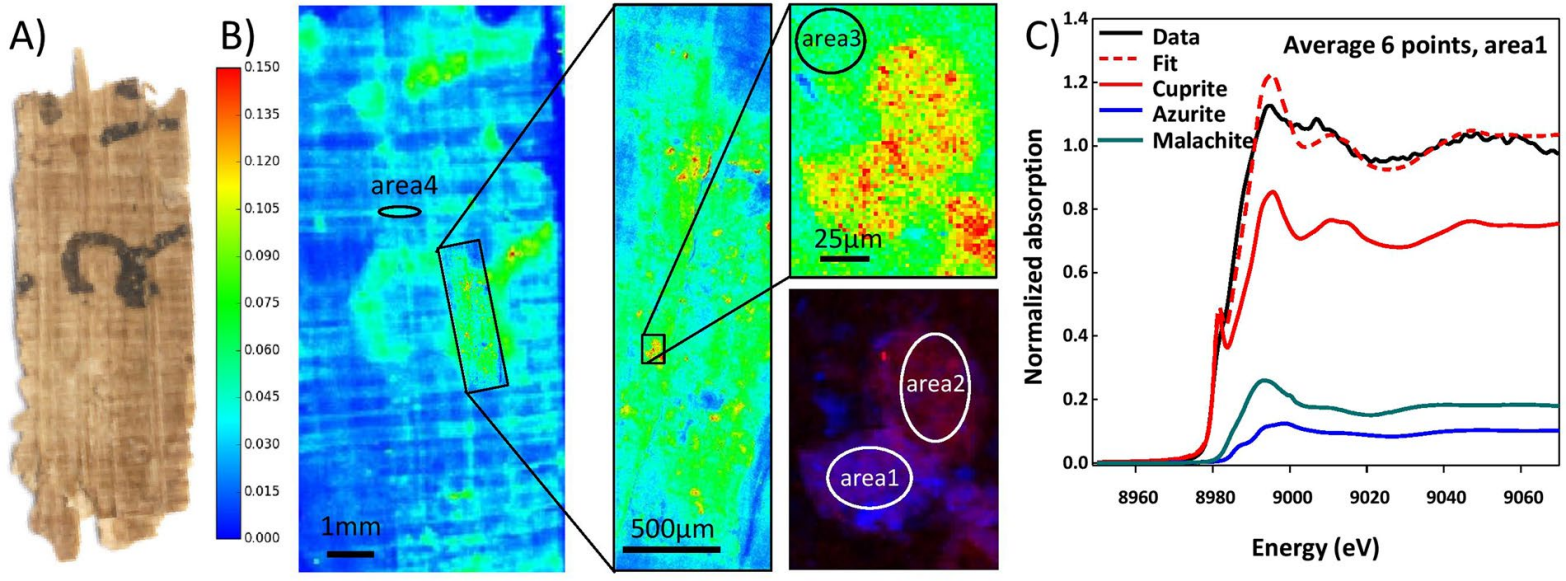
(**A**) Visible light picture of sample 4 (P. Carlsberg 649) (**B**) macro and micro XRF maps of Cu (fitted and normalized by the intensity of incident beam). The areas were XANES spectra were collected are highlighted. The red-blue maps are the superimposition of Cu and Fe maps, from the detailed map (**C**) Average XANES spectra from area 1, and its decomposition by LCF.

Samples 1 and 2 are Greek contracts from Pathyris that date to 134 BCE (Fig. 2A) and 101 BCE (Fig. 3A) respectively. Sample 5 belongs to the same archive and is written demotic, a cursive ancient Egyptian script; it dates to c. 100 BCE. Samples 3 and 4 were found at Tebtunis; they are written in Demotic and can be dated to the 1^st^/2^nd^ century CE (Figs 4A and 5A) on the basis of paleography. For synchrotron-based analyses, the papyri were analyzed, without any sample preparation: the fragments were maintained between two 4 µm thick Ultralene foils (Spec, Certiprep) and mounted vertically in the X-ray microscope.

The black inks on the analyzed fragments appear black at an IR illumination of 970 nm and show no signs of transparency, as observed for other black pigment materials such as iron-gall ink^6–8^. This suggests that the inks used are based on amorphous carbon obtained through the pyrolysis or macerating of botanicals, which is confirmed by Raman spectroscopy carried out on the same papyri, where the spectra are characterized by two broad bands at ca. 1322 and 1588 cm^−1^, known as D and G bands of carbon materials^7,9,10^.

### Macro XRF and micro XRF

XRF measurements were performed at X-ray microscopy beamline ID21 at the ESRF (Grenoble, France)^11^. By the use of a Si (111) monochromator, the primary beam energy was tuned at the Cu-K edge (8979 eV). For general overview mapping over entire fragments (macro XRF), the beam spot size was defined using a pinhole of 100 or 50 µm diameter. The average beam flux was ~10^9^–10^10^ ph/s during the measurements of the samples. An incident beam flux monitoring pin diode was used continuously to monitor and correct for intensity variations (i_0_). XRF maps were acquired by scanning the sample through the X-ray beam with a single energy of 9.05 keV recording a XRF spectrum at each pixel with an acquisition time of 100 ms. High resolution micro XRF maps were acquired the same way, with a beam focused down to ~0.4 × 0.7µm^2^ using a Kirkpatrick-Baez mirror system. The microscope was operated in vacuum and samples were mounted vertically under an angle of 62° with respect to the primary X-ray beam. No visible modification of any aspect of the samples was observed after analysis. The XRF (and scattered) radiation was detected using a Bruker (Germany) XFlash 5100 silicon drift detector (SDD), equipped with a Moxtex AP3.3 polymer window^12^, and mounted under 69° with respect to the primary X-ray beam. An additional Ultralene foil (4 µm) covered the detector. XRF spectra were processed using the PyMCA software package^13^. Elemental maps shown in the figures below are the batch fitted XRF intensity maps, divided by the i_0_ map.

Micro XRF maps were acquired for sample 2 at three different energies to map the different Cu species. The small beam shift between these different maps was determined using the Fe maps and Cu maps were realigned accordingly, using the “Spectrocrunch” python software library^11^.

### Micro XANES

The measurements were performed at ID21, at the Cu K-edge (calibrated with a Cu foil, setting the maximum of the derivative spectrum at 8.979 keV). Micro XANES spectra were recorded in XRF mode (using the same set-up described above) with a micro beam of 0.4 × 0.7µm^2^. The micro XANES spectra were obtained by scanning the primary energy from 8.9 to 9.15 keV in 260 steps of 0.3 eV. To reduce risks of radiation damage, XANES spectra were acquired as single acquisition (30 s/point) over many points, instead of cumulating many spectra on few points. Normalized data were employed for Linear Combination Fitting (LCF), using the ATHENA software, to identify and to estimate the amount of copper compounds on the analyzed papyrus (cf. list of reference in Table 1)^14^. The reference compounds were prepared as powder and measured in transmission mode. LCF were accomplished within −20 < E_0_ > 30 eV range, using first all the references (azurite, malachite, chalcanthite, tenorite, cuprite, chalcopyrite and Cu acetate), and then reducing this set to the main 3 or 4 components; all amounts between 0–1, but not forced to sum 1 for better alignment (amounts were recalculated); all spectra shared the same E_0_ value.

The micro XANES spectra were compared to selected reference compounds (Table 1), chosen as the most probable compounds according to the micro XRF maps and the available literature^1,15–19^. In general, the LCFs have good R-factors (0.002–0.017). However, it has to be kept in mind that all results below could be biased by this set of references and we cannot exclude that other Cu compounds may be present.

**Table 1:** Results of the LCFs analysis of XANES spectra, calculated as average over n points per area. Areas are located in the different XRF maps (cf. Figs 2B, 3B, 4B and 5B).

Sample Standard	Nb of pts/area	Cu distribution	Azurite Cu_3_(CO_3_)_2_(OH)_2_	Malachite Cu_2_CO_3_(OH)_2_	Cuprite Cu_2_O	Copper acetate Cu(CH_3_COO)_2_	Chalcanthite CuSO_4_·5H2O	Tenorite CuO	Chalcopyrite CuFeS_2_	R-factor
			Cu (II)	Cu (II)	Cu (I)	Cu (II)	Cu (II)	Cu (II)	Cu (I)	
Sample 1 – area 1	6	Ink, high Cu intensity	0.18	0.27	0.55	X	X	X	X	0.0070
Sample 1 – area 2	6	Ink, medium Cu intensity	0.19	0.31	0.55	X	X	X	X	0.0087
Sample 1 – area 3	5	Cu rich spot	0.21	0.23	0.57	X	X	X	X	0.0067
Sample 2 – area 1	10	Cu rich spot	0.71	0.00	0.07	0.22	X	X	X	0.0024
Sample 2 – area 2 WS	3	Cu rich spot WS	0.71	0.12	0.17	0.00	X	X	X	0.0056
Sample 2 – area 2 WOS	4	Cu rich spot WOS	0.43	0.00	0.00	0.57	X	X	X	0.0070
Sample2 - area3 WS	3	Cu rich spot WS	0.54	0.31	0.15	0.00	X	X	X	0.0085
Sample 2 – area 3 WOS	6	Cu rich spot WOS	0.54	0.00	0.00	0.46	X	X	X	0.0056
Sample 2 – area 4 ink	24	Ink, medium Cu intensity	0.57	0.00	0.27	0.16	X	X	X	0.0020
Sample 2 – area 4 fiber	15	Cu in fiber	0.20	0.28	0.52	0.00	X	X	X	0.0070
Sample 2 – area 5 fiber	45	Cu in fiber	0.20	0.26	0.54	0.00	X	X	X	0.0088
Sample 3 – area 1	8	Ink, medium Cu intensity	0.19	0.19	0.63	0.00	X	X	X	0.0104
Sample 3 – area 2	6	Ink, high Cu intensity	0.37	0.08	0.55	0.00	X	X	X	0.0078
Sample 3 – area 3	7	Cu close to, but outside of ink	0.11	0.21	0.68	0.00	X	X	X	0.0132
Sample 4 – area 1	7	Cu and Fe rich spot	0.25	0.15	0.59	X	X	X	X	0.0083
Sample 4 – area 2	6	Cu rich spot	0.10	0.17	0.73	X	X	X	X	0.0138
Sample 4 – area 3	10	Ink, medium Cu intensity	0.04	0.25	0.71	X	X	X	X	0.0170
Sample 4 – area 4	18	Cu in fiber	0.05	0.25	0.70	X	X	X	X	0.0154

WS: with shoulder; WOS: without shoulder. These points were selected on micro XRF maps at three energies (cf. text and Fig. 2B).

## Results

### Macro XRF maps

The papyrus fragments were scanned using X-ray beams of different sizes, from a sub-millimeter to a micrometric scale. Macro-XRF maps of the full fragments were used to identify and localize elements both outside and inside of the ink (cf. the supporting information, where all the XRF elemental maps of the five samples are provided and some specific results are commented).

Although the papyri derive from different time periods and geographical areas, the elemental composition detected in the fragments is similar and showed the following distributions: potassium (K) and chlorine (Cl) maps reveal the fibrous structure of the papyri. Silicon (Si) shows a complementary distribution; as if it fills the holes left by the K-Cl based fiber structure. Sodium (Na), magnesium (Mg), aluminum (Al), phosphor (P), sulfur (S), calcium (Ca) and manganese (Mn) are present in a rather homogeneous way on the surface of the papyrus fragments, independently of the fibrous structure. Iron (Fe) is present as spots all over the papyri, independently of the ink, except for sample 4, where Fe-Al-K containing spots are more concentrated in the inked regions (Fig. S11).

The Cu elemental distribution (fitted XRF intensity divided by the intensity of incoming beam) for samples 1, 2, 3 and 4 is depicted in Figs 2B, 3B, 4B and 5B. The color scales are identical for the large map of samples 1, 2 and 3 (0–0.5, a.u.). Because sample 4 shows lower amounts of Cu in the ink, the scale has been adjusted to 0–0.15 a.u. in Fig. 5. From the maps, it is clear that the copper is concentrated in the letters and signs from where it diffuses out in the papyri and runs along the fibrous structure.

As seen in the supporting information, some Cr maps show a peculiar circular structure that is due to the sample holder. This demonstrates that the X-rays penetrated the full depth of the samples (Figs S5, S6, S8).

### Micro XRF maps

Additional XRF maps were acquired on selected areas with Cu-containing black ink, in order to assess at the micrometer scale the possible co-localization of certain elements that were detected at the macro-scale.

Sample 1: as observed in the macro-XRF maps, Cl, K and Cu maps show some correlations, but this may be due to the strong diffusion of Cu in the K-Cl fibrous structure. The other elements do not show a particular co-localization with Cu (Fig. S4).

Sample 2: a high resolution micro XRF map revealed significant variation in the distribution of Cu both within and outside the ink area. To further investigate these differences, five areas were examined more closely, encompassing the ink, the surrounding fibers and Cu-rich spots (Figs 3B, S6 and S7), with a pixel size of 1 or 2 µm. None of the other detected elements were co-localized with Cu. As an example, a Fe map is shown in Fig. 3C.

Sample 3: micro XRF maps confirmed the results obtained from the macro XRF maps (cf. Fig. S8), which showed slightly higher counts of Mg, Al, S, P, Ca, Mn and Pb in the ink than in the papyrus (Fig. S9). The detailed maps show a similar co-localization of Cu with P, S and Pb at the micron-scale. However, considering the diffuse distribution of these elements and the absence of micrometric Cu-based spots, it cannot be concluded that these elements originate from the copper source; they are rather associated with the soot and the binder (Fig. S10).

Sample 4: spots containing Mg, Al, Si, P, K, Cr, Mn and Fe are found, but they are not co-localized with Cu (cf. Figs 5B, S11 and S12). Other spots contain Ca and S, Ca and P, or Ca alone. S, Cl and Ca were more concentrated in ~50 µm Cu-rich regions (Fig. S11), but without co-localization to Cu at the micron-scale (Fig. S12).

### Micro XANES

In order to examine the Cu speciation and possibly its origin in the Cu-rich inks, Cu K-edge micro XANES spectra were acquired at different points of the four samples. The fact that none of the other detected elements were co-localized with Cu led us to the assumption that Cu was most probably present as elements from the first and second periods of the periodic table, e.g. oxides, hydroxides, carbonates or organic salts.

For sample 1, we recorded micro XANES spectra at 17 points located in three different regions highlighted in Fig. 1C. Since the spectra were very similar, they were subsequently averaged. The LCF of the average of Cu-K XANES spectra was done using the above mentioned reference compounds, excluding chalcopyrite, since Cu is not co-localized with Fe. In the three areas, micro XANES spectra show features characteristic of a mixture of Cu^1+^ (fitted as cuprite) and Cu^2+^ species (fitted as azurite and malachite) (Table 1 and Fig. 2C).

A total of 120 spectra were collected for sample 2 in the five areas shown in Fig. 3C. These spectra showed clear differences from one area to another, but also within a single Cu spot. As an example, some of the spectra acquired over the spots in area 1, 2 and 3 showed a pronounced shoulder at ~8.987 keV, while this shoulder was mostly absent in spots at other locations. To map the distribution of these different species, speciation maps were acquired by collecting micro XRF maps at three different energies: at the shoulder energy (E_1_ = 8.987 keV), at the maximum absorption energy (E_2_ = 8.997 keV) and above the edge – in order to map the Cu distribution, independently of its speciation (E_3_ = 9.075 keV). The superimposed Cu maps obtained at E_1_ and E_2_ are shown in Fig. 2B and reveal a tight interlacing of the two Cu-based ingredients. None of these maps is correlated with the Fe map (Fig. 3B). The LCF of the average spectra exhibiting a strong shoulder at E_1_ gives azurite as the main component (the azurite reference contains a pronounced shoulder at E_1_), together with some malachite and cuprite (Table 1 and Fig. 3C). The spectra with a less intense shoulder could be fitted with a strong contribution of copper acetate, with smaller amounts of azurite (Table 1 and Fig. 3C). Spectra acquired in area 4 – i.e. in the ink, but not in the ‘spotty’ Cu regions – were fitted as azurite mainly, together with cuprite and copper acetate. Finally, the spectra acquired in regions where Cu has diffused within papyrus fibers, but outside areas of actual writing, show a lower signal but with a more pronounced shoulder at 9.075 keV, together with a clear shift of the edge energy. These features could be fitted by a high contribution of cuprite (>50%), mixed with malachite and azurite (Table 1 and Fig. 3C).

For sample 3, a total of 21 spectra were acquired: six in the ink in the region with high Cu content, eight in the ink with lower Cu content and seven spectra in a region, where the Cu distribution follows the fibrous structure. The LCF analysis of the average spectra gives cuprite as the main component, together with azurite and malachite (Table 1 and Fig. 4C).

A total of 41 spectra were acquired for sample 4 over four areas (Fig. 5): in spots with a high content of Cu both with and without Fe, in ink regions with lower Cu content and in fibrous Cu rich regions, far removed from the ink. In all these areas, cuprite is the main component of the LCFs. Malachite is the second component, and azurite to a lesser extent (Table 1 and Fig. 5C)

## Discussion

The synchrotron based macro and micro XRF maps confirmed the presence of Cu in the black ink on the four ancient Egyptian papyri studied here. In sample 2, 3 and 4, the ink contains Cu and other lighter elements – Al, Si, K, Mn, Fe – and Pb. However, the study of Cu spots at the micron-scale did not reveal any clear local co-localization of these elements with Cu.

Micro XANES revealed that Cu in inked areas is present principally as the copper minerals cuprite, azurite and malachite. In Egypt these minerals are present along almost the entire length of the eastern desert and in the Sinai, and their use in the production of green and blue pigments has been amply documented^18,20^.

In the areas where Cu is diffused into the fibrous structure of the papyri, and in the complex Cu-rich spots in sample 2, malachite occurs as one of the components. It may be present as part of the original pigment or be formed as a result of the degradation of azurite^18^. The copper acetate present in sample 2 could also be a result of a reaction of the copper minerals with the chemical compounds in the surroundings or reactions caused by the conservation procedures.

Ancient copper-containing pigments are well-known as a source of the catalyzed degradation of cellulose based materials such as gum-Arabic and papyrus;^16^ for instance Egyptian blue and green can ‘burn’ holes in illustrated papyrus manuscripts^20^. A degraded binder (gum-Arabic) could explain why the inks visually appear to be ‘cracking’ and the diffuse presence of Cu outside the letters and signs in the fibrous structure of the papyrus. Likely, the migration of the Cu along the fibers was enhanced by the conservation procedures applied to the manuscripts. There is no detailed documentation on the method of conservation applied to the fragments, but it usually consists of a simple process, where the papyri were moistened with water in order to relax the fibers and unfold or unroll them; thereafter, they were mechanically cleaned with a sharp instrument and a sable brush^21,22^. With respect to cuprite (Cu_2_O) it could be a result of a reduction of copper carbonate pigments like azurite and malachite, the two other principal Cu compounds detected in the ink and along the fibrous structure of the four fragments^17^. However, there is also evidence that the cuprite present in the ink could have undergone an oxidation reaction in the presence of water and CO_2_ in the atmosphere, which transversely would lead to the formation of azurite and malachite^16^.

These observations suggest that the source of the Cu compounds found in the black inks and along the fibrous structure are by-products of metallurgy, glaze and glass production, which provided the raw material (soot) for “refined” carbon inks in the ancient Mediterranean. This is supported by the few preserved written formulae from the Hellenistic Period pertaining to the manufacture of black ink^1^.

## Conclusion

Looking at the results, it is likely that the soot/charcoal of copper-containing carbon inks were obtained during manufacturing processes related to the extraction of copper from sulfurous ores like chalcopyrite. This hypothesis finds confirmation in the particle size (sub-micron) and the fact that another copper-bearing pigment in Egypt, the so-called Egyptian blue (CaSi_2_O_5_·CuSi_2_O_5_), was manufactured from scrap or by-product copper obtained at temple workshops that either melted copper or produced glass and faience^23^. It was made by mixing cupric oxides with sand, soda and lime, which thereafter was roasted at about 850–900 °C to sintered crystalline aggregates rather than glass^18,24–26^. Similarly, Egyptian kohl or black eye-paint, which is closely related to the manufacture of lead-containing carbon inks, was produced in workshops, where vitreous materials were manipulated^27^.

Since the papyri in question were written over a period of 300 years, the findings cannot represent an accidental event. Moreover, sample 1 is the oldest dated document from the ancient Mediterranean in which the addition of metals to a black ink has been detected. Though the fabrication of ink is likely to have evolved during this time-span, none of the four inks studied here are completely identical and Cu micro XANES showed variations within a single fragment. This demonstrates a variable local ink composition, and by extension production, which precludes the chance to obtain unique signature of the ink based on Cu speciation. This observation complicates the mapping of inks, but might facilitate the identification of fragments belonging to specific manuscripts or sections thereof^7^. Moreover, it should be taken into account that Cu speciation may have evolved since the original preparation and use of the ink. In particular, conservation treatments may have modified the Cu chemistry. Finally, the results will facilitate future strategies of conservation, since knowledge of material composition assists decisions, which remain to be made regarding the proper conservation and storage of the papyri, thereby ensuring their preservation and longevity. Why and when copper-containing carbon inks were introduced in ancient Egypt remain to be explained, but perhaps it is related to the type of pen used for writing the manuscripts, since the four papyri appear to have been written with a Greek reed pen (*kalamos*) rather than an Egyptian reed brush^28^.

## Electronic supplementary material

Supporting Information
