# Supplementary material for: The nature of ancient Egyptian copper-containing carbon inks is revealed by synchrotron radiation based X-ray microscopy
Source: Sci Rep. 2017 Nov 10;7:15346. doi: 10.1038/s41598-017-15652-7 (PMC5681681; doi:10.1038/s41598-017-15652-7)
Supplement: Supplementary file 1 — Supporting Information [file 41598_2017_15652_MOESM1_ESM.pdf]

SUPPORTING INFORMATION

The nature of ancient Egyptian copper-containing carbon inks is revealed by synchrotron radiation based X-ray microscopy

Thomas Christiansen, Marine Cotte, René Loredo-Portales, Poul Erik Lindelof, Kell Mortensen, Kim Ryholt, Sine Larsen

X-ray fluorescence elemental maps

Figure S1 shows visible light pictures and macro XRF elemental maps of a papyrus fragment (P. Carlsberg 860) dating to c. 100 BCE, which was found at Gebelein and acquired through the antiquities markets in 1924 (sample 5). The inked area cannot be discerned in the maps of the excited elements, indicating that the pigment is composed of amorphous carbon. The figures S2-12 present the macro and micro elemental maps that correspond to those shown in Fig. 2-5 of the article.

The maps have been obtained from the XRF maps, after normalizing the XRF intensity by the i_0 map and performing batch fitting with the PyMCA software. The spectral resolution of the silicon drift detector (SDD) detector ranges from ~ 120eV at 1keV to 180eV at 8 keV and is sufficient for distinguishing the different fluorescence lines (cf. Fig 1). The maps are displayed in a temperature color scale, where blue is min and red is max. By default the color scale covers the complete min-max range, except in cases, where some hot spots were less intense in their distribution (in particular for Cu). In such cases, the max value of the color scale has been reduced to better highlight these structures.

In addition to the outcomes listed in the article, the following specific results were observed in relation to the macro XRF elemental maps:

Sample 1: Mg and Al exhibit regions of diffuse higher amounts, but are not correlated with the ink. Cl, K and Cu maps show some correlations, but this may be due to the strong diffusion of Cu in the K-Cl fibrous structure (Fig. S2 and S3).

Sample 2: Cu is homogeneously distributed in the ink and diffused in the fiber structure of the papyrus. Further, it is also present as ‘spotty’ particles around the inked areas. Mg, Al, Si, P, S, Cl, K, Mn and Pb are slightly more concentrated in the ink (Fig. S5).

Sample 3: Mg, Al, P, S, K, Ca, Mn and Pb show a slightly higher concentration in the ink (Fig. S9). Ca and Mn are also diffusely concentrated in areas outside of the ink. Na and Cl are co-localized in large particles (>40µm). Other particles contain K and Fe. These particles are not correlated with ink.

Sample 4: Mg, Al, Si, P, S, Cl, K, Ca, Cr, Mn, Fe and Co are slightly more concentrated in the ink (Fig. S5).

It should be noted that in some Cr maps a peculiar circular structure - due to the sample holder – can be seen (Fig. S5, S6, S8 and S9).

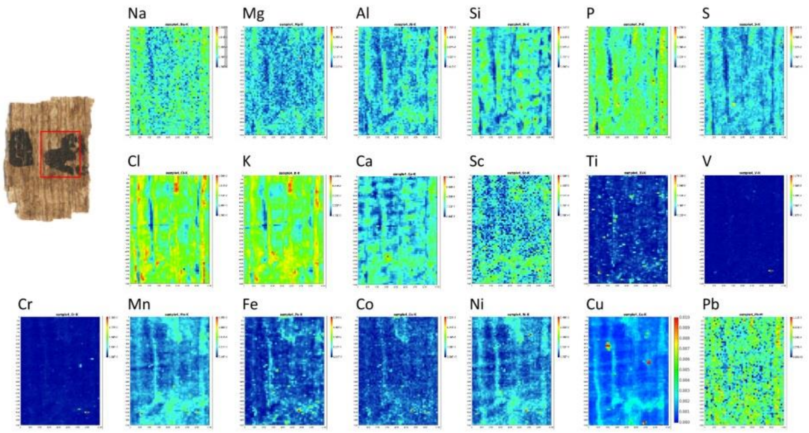

Figure S1: Visible light pictures and macro XRF elemental maps of sample 5. The red insert marks the area scanned with a unfocused beam (52×70 steps of 80μm).

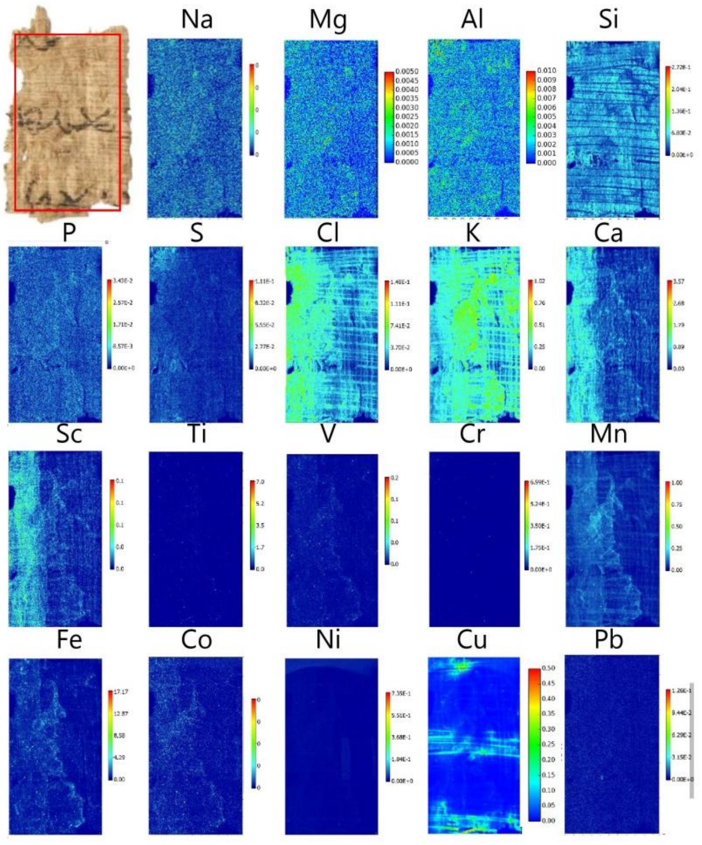

Figure S2: Visible light pictures and macro XRF elemental maps of sample 1 (cf. Fig. 2). The red insert marks the area scanned with an unfocused beam (404×214 steps of 50μm).

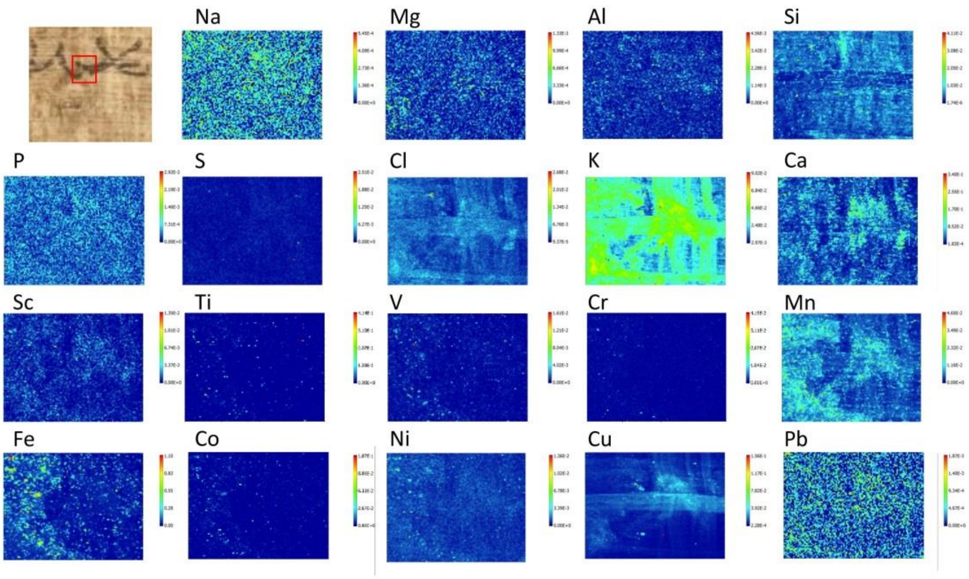

Figure S3: Visible light pictures and macro XRF elemental maps of sample 1 (cf. Fig. 2). The red insert marks the area scanned with an unfocused beam (108×83 steps of 20µm).

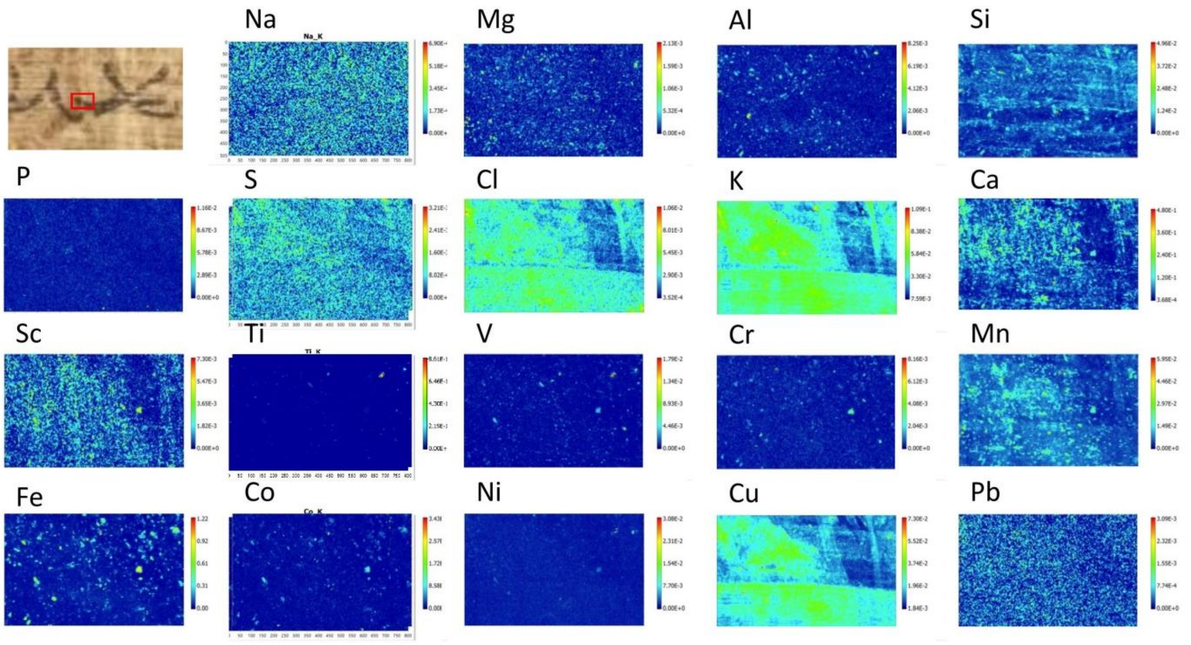

Figure S4: Visible light pictures and micro XRF elemental maps of sample 1 (cf. Fig. 2). The red insert marks the area scanned with a focused beam (160×100 steps of 5µm).

Figure S5: Visible light pictures and macro XRF elemental maps of samples 2 (left) and 4 (right) (cf. Fig. 3 and Fig. 5). The red insert marks the area scanned with a unfocused beam (253×244 steps of 50µm).

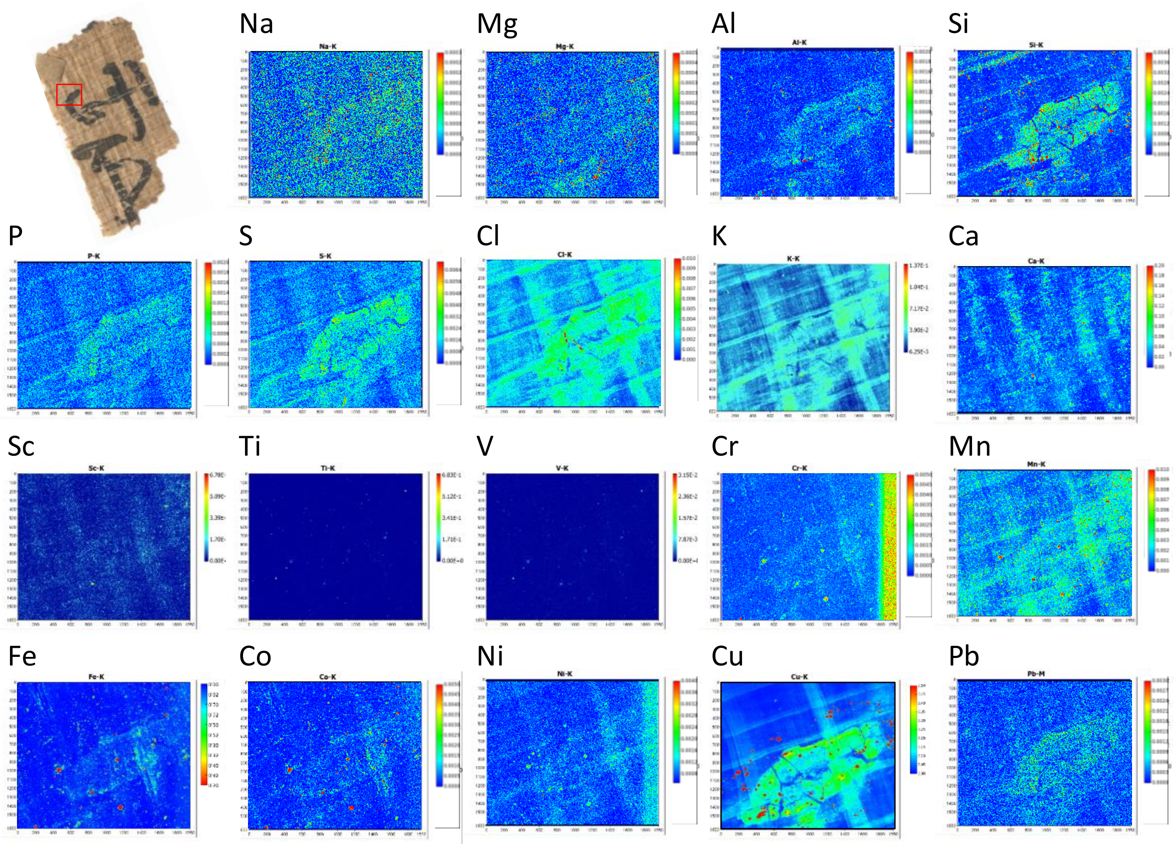

Figure S6: Visible light pictures and micro XRF elemental maps of sample 2 (cf. Fig. 2). The red insert marks the area scanned with a focused beam (390×330 steps of 5µm).

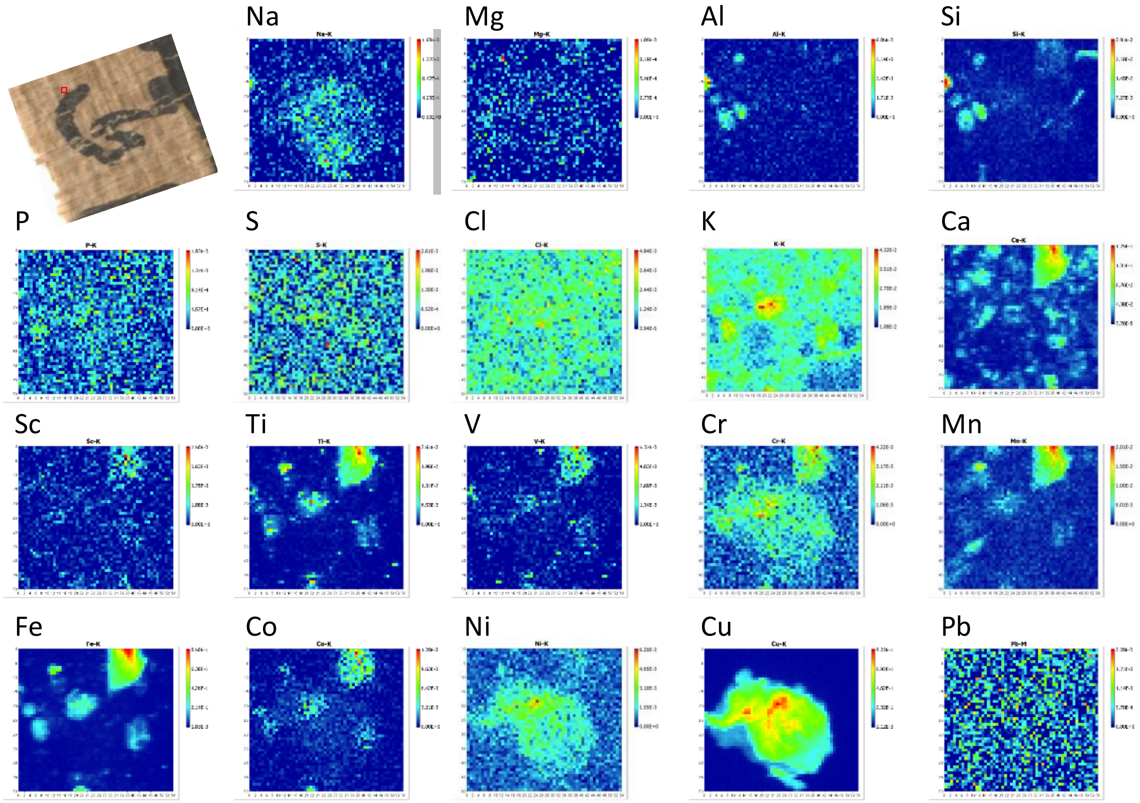

Figure S7: Visible light pictures and micro XRF elemental maps of sample 2 (Cf. Fig. 2). The red insert marks the area scanned with a focused beam (59×49 steps of 1μm).

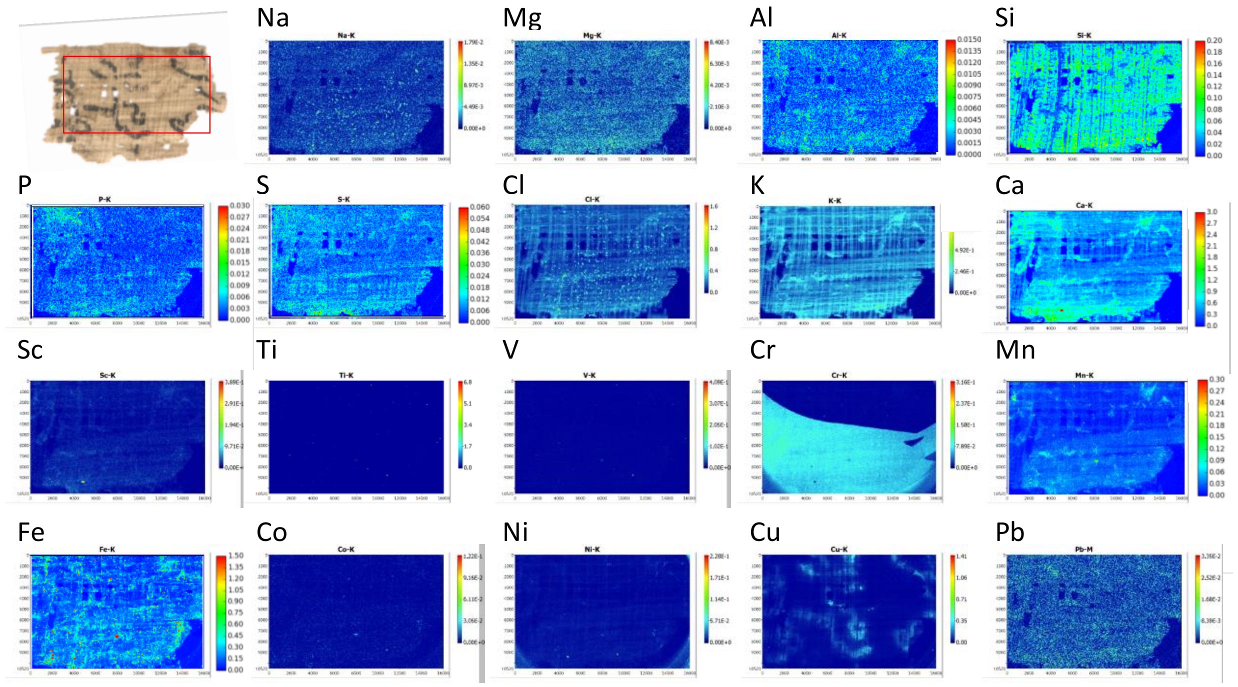

Figure S8: Visible light pictures and macro XRF elemental maps of sample 3 (cf. Fig. 3). The red insert marks the area scanned with an unfocused beam (400×262 steps of 50µm).

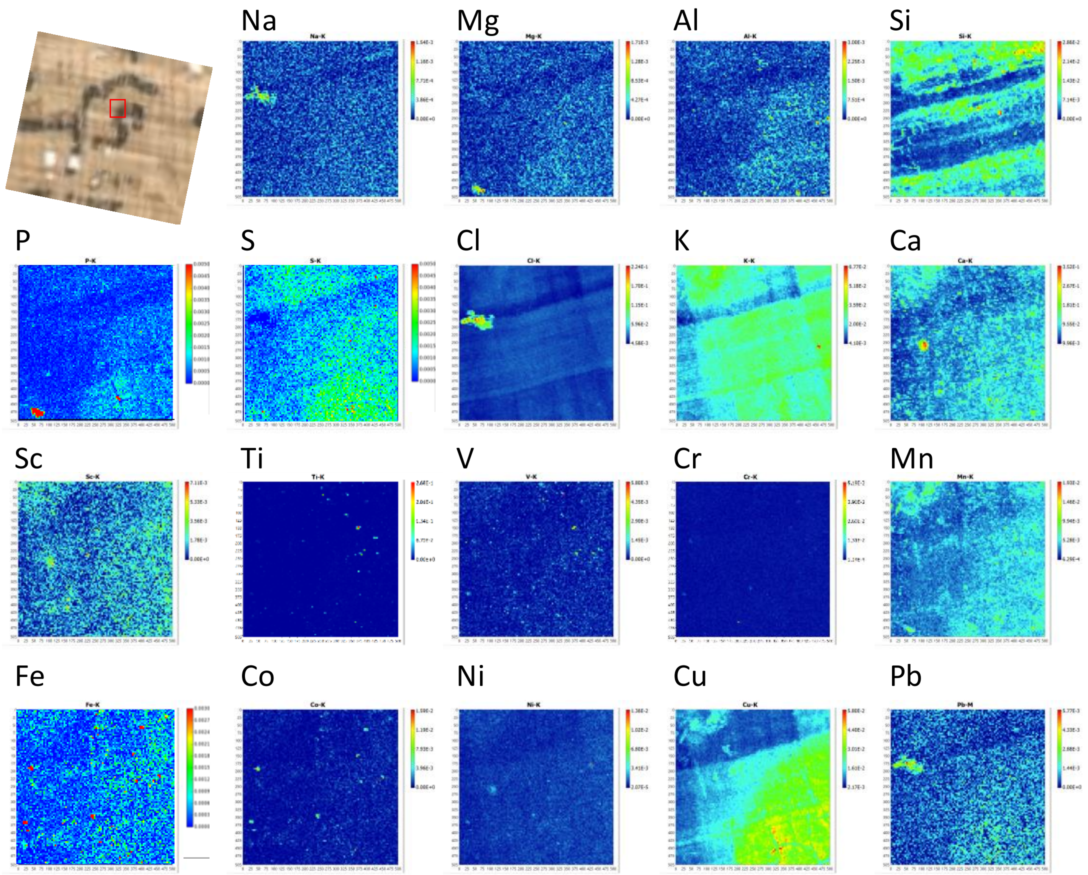

Figure S9: Visible light pictures and micro XRF elemental maps of sample 3 (cf. Fig. 3). The red insert marks the area scanned with a focused beam (100×100 steps of 5µm).

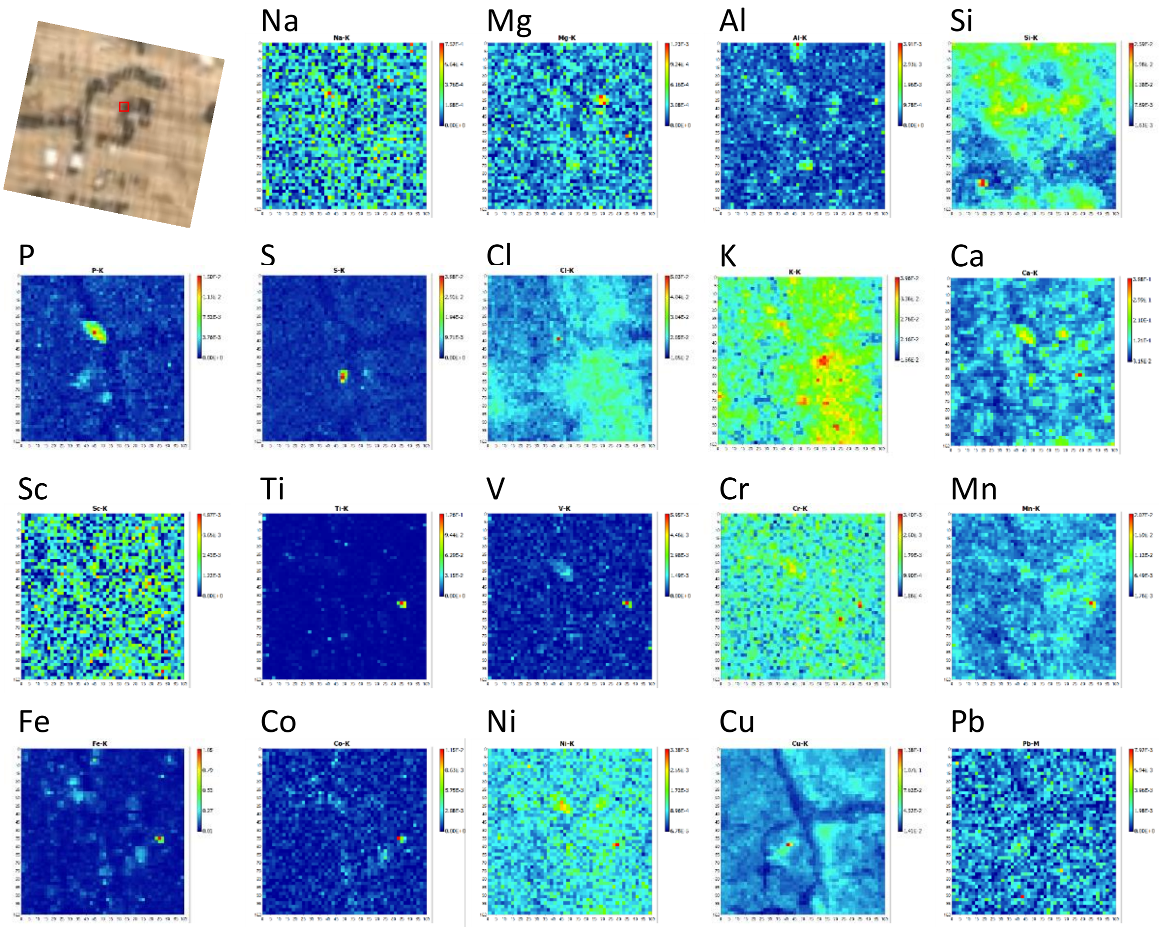

Figure S10: Visible light pictures and micro XRF elemental maps of sample 3 (cf. Fig. 3). The red insert marks the area scanned with a focused beam (50×50 steps of 2µm).

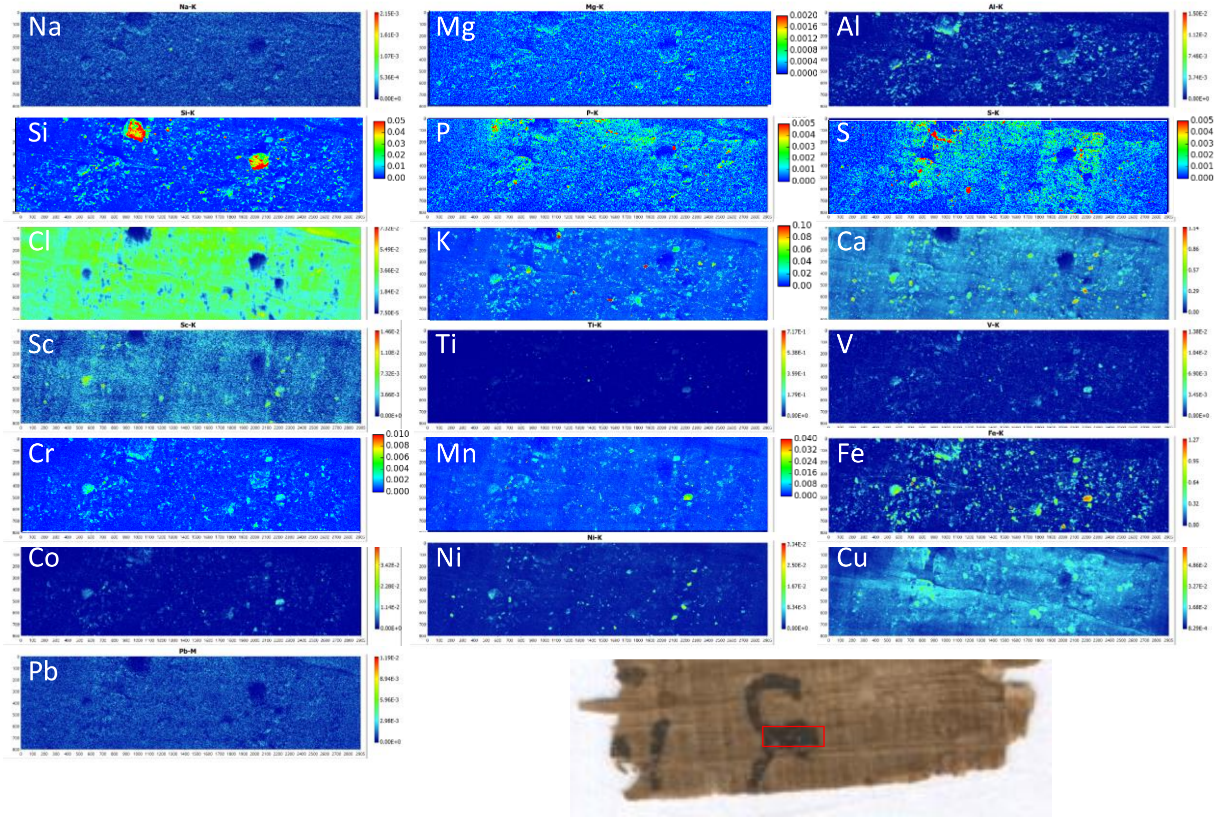

Figure S11: Visible light pictures and macro XRF elemental maps of sample 4 (cf. Fig. 4). The red insert marks the area scanned with a focused beam (581×159 steps of 5µm).

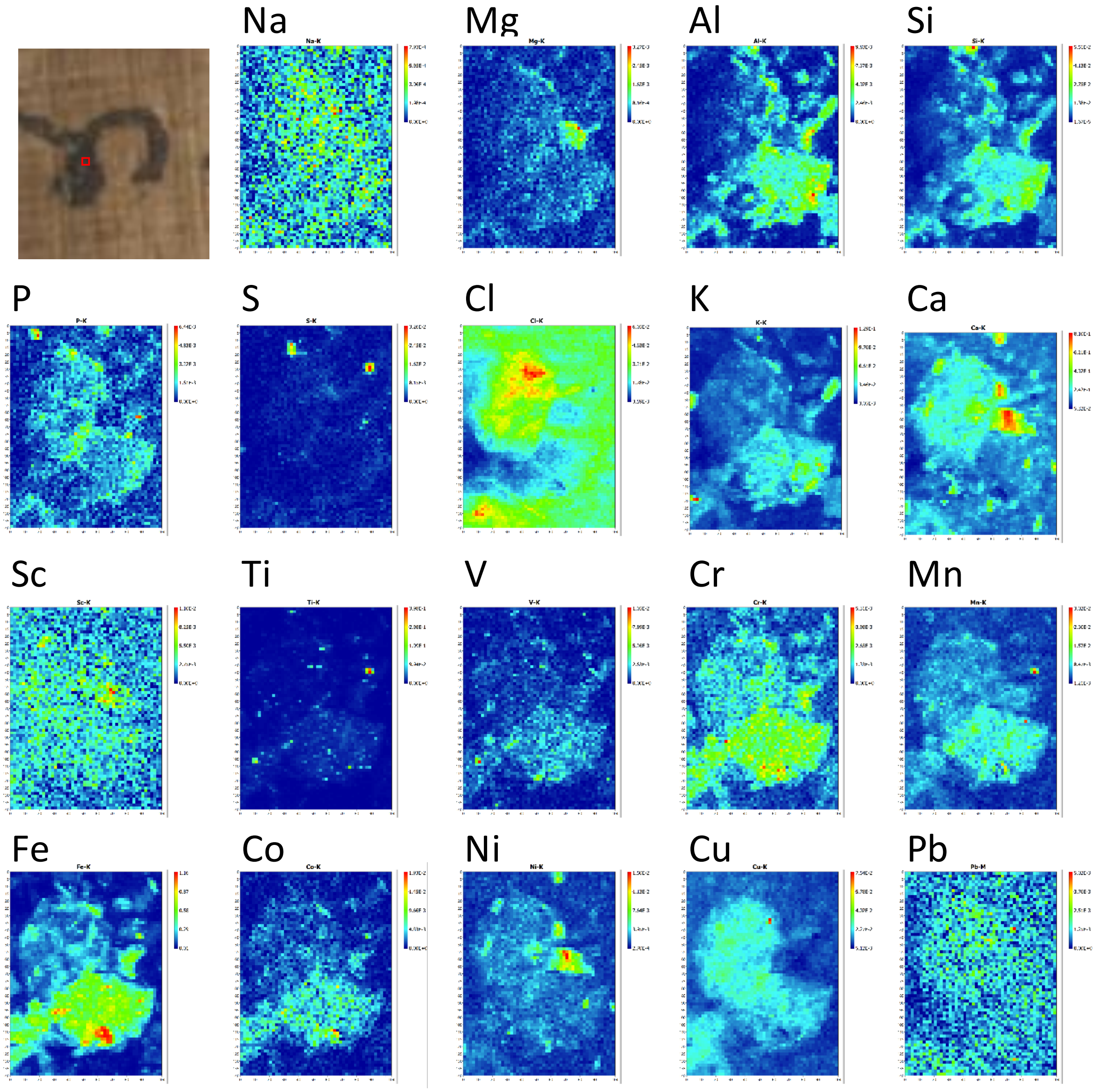

Figure S12: Visible light pictures and micro XRF elemental maps of sample 4 (cf. Fig. 4). The red insert marks the area scanned with a focused beam (52×69 steps of 2µm).

References

[1] Solé, V.A., Papillon, E., Cotte, M., Walter, P., Susini, J. (2007) A multiplatform code for the analysis of energy-dispersive X-ray fluorescence spectra. Spectrochimica Acta Part B: Atomic Spectroscopy **62** (1): 63–68, doi: 10.1016/j.sab.2006.12.002
